# Foot Strike Angle Prediction and Pattern Classification Using LoadsolTM Wearable Sensors: A Comparison of Machine Learning Techniques

**DOI:** 10.3390/s20236737

**Published:** 2020-11-25

**Authors:** Stephanie R. Moore, Christina Kranzinger, Julian Fritz, Thomas Stӧggl, Josef Krӧll, Hermann Schwameder

**Affiliations:** 1 Department of Sport and Exercise Science, University of Salzburg, Schlossallee 49, 5400 Hallein/Rif, Austria; stephanie.moore@sbg.ac.at (S.R.M.); thomas.stoeggl@sbg.ac.at (T.S.); josef.kroell@sbg.ac.at (J.K.); 2 Salzburg Research Forschungsgesellschaft m.b.H., Jakob-Haringer-Straße 5, 5020 Salzburg, Austria; christina.kranzinger@salzburgresearch.at; 3 Adidas AG, Adi-Dassler-Strasse 1, 91074 Herzogenaurach, Germany; julian.fritz@adidas.com; 4 Athlete Performance Center, Red Bull Sports, Brunnbachweg 71, 5303 Thalgau, Austria

**Keywords:** decision tree, human running, random forest, regression, wearable devices

## Abstract

The foot strike pattern performed during running is an important variable for runners, performance practitioners, and industry specialists. Versatile, wearable sensors may provide foot strike information while encouraging the collection of diverse information during ecological running. The purpose of the current study was to predict foot strike angle and classify foot strike pattern from Loadsol^TM^ wearable pressure insoles using three machine learning techniques (multiple linear regression―MR, conditional inference tree―TREE, and random forest―FRST). Model performance was assessed using three-dimensional kinematics as a ground-truth measure. The prediction-model accuracy was similar for the regression, inference tree, and random forest models (RMSE: MR = 5.16°, TREE = 4.85°, FRST = 3.65°; MAPE: MR = 0.32°, TREE = 0.45°, FRST = 0.33°), though the regression and random forest models boasted lower maximum precision (13.75° and 14.3°, respectively) than the inference tree (19.02°). The classification performance was above 90% for all models (MR = 90.4%, TREE = 93.9%, and FRST = 94.1%). There was an increased tendency to misclassify mid foot strike patterns in all models, which may be improved with the inclusion of more mid foot steps during model training. Ultimately, wearable pressure insoles in combination with simple machine learning techniques can be used to predict and classify a runner’s foot strike with sufficient accuracy.

## 1. Introduction

Recreational running is a globally accessible activity due to the limited necessity of sport-essential equipment and facilities. Due to its full-body nature, the human anatomical system has many ways to affect running performance. Some factors of paramount importance are joint angles (which thus affect stride length), flight time, and the minimization of lateral force-dissipation [1,2]. The selection of the running shoe also appears to affect performance [3,4], the subjective experience of comfort [5,6], and the injury risk of runners [3,7]. Equipment-based recommendations should include the consideration of a runner’s foot strike pattern (FSP) [8]. A midsole design that facilitates the repetitive and comfortable execution of the preferred FSP (i.e., rear foot (RF), mid foot (MF), or fore foot (FF)) can aid the consumer-based shoe selection and recommendation process [3]. Such a recommendation thus requires a reliable method for the discrete classification of a runner’s FSP as a prerequisite.

Some performance-related outcome variables are affected by the FSP used, including the vertical compliance of the anatomical system [9], ankle and knee stiffness [10], vertical impact force [11], and instantaneous loading rates [11]. Importantly, these variables can be measured on a continuous scale and are likely responsive to more sensitive foot strike angle (FSA) measurements. More specifically, the FSA is the angular degree of the foot at the instant of ground contact (often defined by a force or loading rate threshold) [12,13]. Therefore, the ability to detect the degree of foot strike on a continuous level enables greater correlational insights that may be overlooked by a discrete classification-based system [14]. Thus, in addition to the necessity of FSP classification, the continuous-scale identification of a runner’s FSA should be accessible for researchers and performance-centered practitioners.

With the growing importance for ecologically valid shoe prescription and scientific investigation of runners, the ability to detect and classify the FSA of a runner using wearable sensors is essential. Inertial measurement units (IMUs) are a viable and validated option for ecological FSA collection [8,15,16], although the calculated angular displacements are prone to poor reliability due to drifts over time which thus affect the integration of the inertial signals in IMU systems [17]. The combination of inertial, gyroscopic, and magnetometer information that an IMU provides helps in the reduction and correction of its measured drift, though the rigidity and alignment of the sensor attachment also directly influence the reliability of the angular measures [17]. Alternative to IMU systems, the holistic pairing of kinetic information with the kinematic measurement of FSA from a single measurement system may enable greater insights about performance and injury indicators in running. Thus, a simple, “low-friction” wearable device that could validly provide this holistic view would be groundbreaking for the running industry.

In an effort to fill this innovative gap, the accelerometer-based Stryd^TM^ foot pod attempts to provide this holistic view of the kinematic and kinetic information by estimating running power [18,19,20]. However, Stryd^TM^ appears to have limitations when detecting temporal variables [20]. From a methodological context, running power calculations that require both kinetic and kinematic inputs appear to have better prediction performance of a linear power-velocity relationship than those using kinematic data only [21]. Unfortunately, single IMU-based estimations of ground reaction force (GRF) come with substantial limitations; (i) the placement of an IMU can affect GRF estimate accuracy, (ii) magnetic disturbances can affect the orientation of the IMU, and (iii) the existence of kinematic estimate errors would be inherent in subsequent GRF estimates [22,23]. Thus, a wearable kinetic system may be better equipped to provide this holistic view. 

The wearable application of pressure insoles already extends to temporal gait events [24,25], therefore they may be a plausible alternative to IMUs to facilitate ecological kinematic estimation while also enabling a valid measurement of vertical force (Fong et al., 2008). Importantly, Loadsol^TM^ wearable insoles can measure vertical force in the rear and fore foot separately; thus the time and force relationships of the fore and aft sensors may provide enough information for FSA prediction and FSP classification [26,27]. Further, the separation of the insole into multiple components is encouraged by the assumption that the foot is not a singularly rigid segment as was traditionally considered [28,29]. The Loadsol^TM^ has been previously validated under running stimulus [13,30,31], therefore it is an appropriate system to establish the potential for the kinematic estimation of FSA and FSP.

Machine learning techniques may enable the estimation of FSA and FSP from pressure sensors; these are practical tools that can be trained and implemented into large-scale problems and data sets, with the inherent goal being to capitalize on the distinctive qualities of the data set [32]. Due to a linear relationship between strike index (the percent of foot length at which the center of pressure exists) and FSA [14,16], the linear approach of a multiple regression may be appropriate for the prediction of FSA [27]. In contrast to linear regression models that are based on numerous assumptions (e.g., normality of residuals, homoscedasticity, etc.) [33], nonparametric models such as conditional inference trees or random forests, need only the assumption that similar inputs lead to similar outputs [32]. The prediction and classification accuracy may thus be greater with nonparametric frameworks. Conditional inference trees are a non-parametric class of regression trees that allow for unbiased variable selection and do not require pruning based on resampling [34]. They are based on conditional inference procedures for testing independence between response and each input variable [34]. Alternatively, robust random forest frameworks encourage accuracy gains with the development of multiple variable-randomized trees [35,36].

Ultimately, to confirm best-practice recommendations, the prediction and classification of foot strike using kinetic sensors should be approached from distinctly different statistical techniques. Thus, the purposes of the current study were to compare the accuracy and precision of i) continuous FSA prediction and ii) FSP classification as calculated from three statistical methodologies (multiple regression, conditional inference tree, and random forest) using independent variables derived from the Loadsol^TM^ pressure insoles.

## 2. Materials and Methods

### 2.1. Participants and Experimental Approach

Thirty injury-free recreational male runners (Mean ± SD; 1.79 ± 0.07 m; 80.1 ± 9.6 kg; 34.0 ± 6.9 yr) provided written informed consent approved by the institutional review board to participate in the study. Participants appeared for one testing occasion where they were asked to perform over-ground running using six types of FSPs at a comfortable speed (average velocity = 2.69 ± 0.40 m∙s^−1^). The first condition investigated was their natural running pattern (NA; no constraints), followed by extreme-FF, FF, MF, RF, and extreme-RF in a randomized counterbalanced order. The extreme-FF and extreme-RF conditions were instructed by asking the participants to over-exaggerate their performance of the FF and RF conditions, respectively. Participants were not given any condition-based feedback. All trials were performed with participants running back-and-forth (i.e., shuttle-wise) in a laboratory environment; participants ran a straight distance (5 m) over a force platform located in the center of the straight phase. Participants then quickly changed direction before running the same straight phase. For each participant, 20 non-consecutive left foot-fall instants were recorded per foot strike condition (n = 120 steps). Participants were allowed 5–10 minutes for a self-selected running warm up and familiarization laps were performed before each condition until the participants expressed comfortability with the desired foot strike condition. Importantly, the measured foot falls were labelled as their true pattern or angle, regardless of the condition in which it was performed (see subsequent sections). However, the consistency of participant’s performance of the FSA was assessed in a supplementary analysis which boasted generally good consistency [37].

### 2.2. Measurements

**Figure 1:**
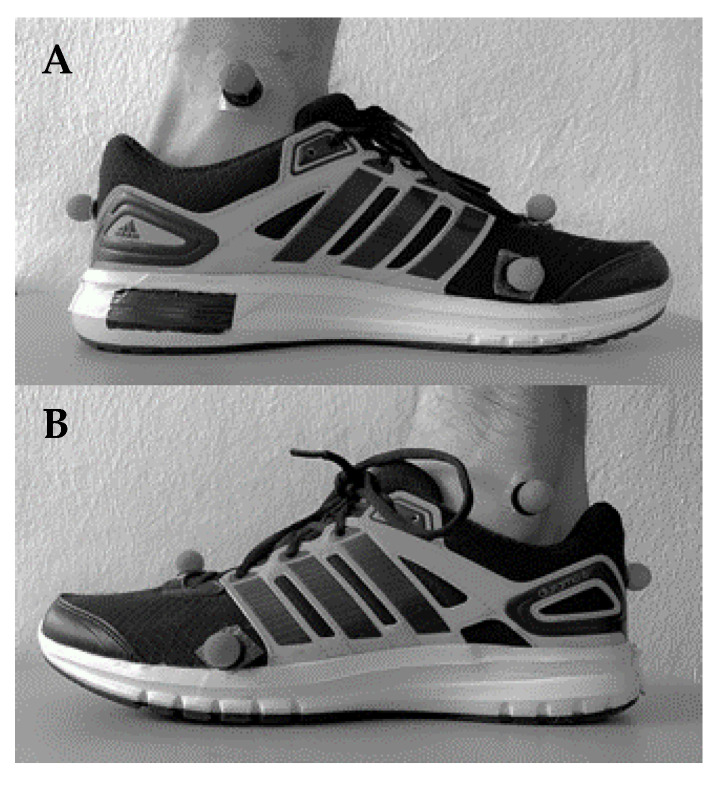
A medial view (**A**) and lateral view (**B**) of the left foot marker placements can be seen on the test shoe.

Insole pressure, force plate kinetics, and kinematics were recorded for 3,489 foot falls (originally, 120 steps per participant × 30 participants = 3,600 foot falls; however only 3,489 are reported due to collection error or data loss). The pressure measurements were achieved with a two-sensor (fore-aft) wireless insole (Loadsol^TM^; Novel GmbH; Munich, Germany) inserted into standardized shoes worn by the participants (Adidas Duramo 6; weight = 280 g., heel drop = 11 mm). The Loadsol^TM^ system was applied over the shoe’s insole and recorded at its maximum sampling rate (100 Hz). Kinetic data from a force platform (AMTI; Watertown, MA, USA; BP6001200) and three-dimensional (3D) motion capture was recorded with a Qualysis system (13-camera setup; 2019.3, Göteborg, Sweden) and sampled at 100 Hz to match the maximum sampling rate of the Loadsol^TM^ system. A six-marker anatomical marker set was applied to the left foot segment (over the shoe when necessary); retroreflective markers were secured on the medial and lateral malleoli, the head of the 2nd metatarsal, the heel (placed at the same height as the 2nd metatarsal), the medial side of the 1st metatarsal, and the lateral side of the 5th metatarsal (Figure 1A,B) [38]. The kinematic and Loadsol^TM^ data were synchronized by aligning the peak force of a stomp measured by the AMTI force platform (data logging with Qualysis) and Loadsol^TM^ at the beginning of each trial.

### 2.3. Data Processing 

Initial contact (IC) and toe off (TO) were identified from the Loadsol^TM^ measurements as the frame in which the loading rate of the pressure insoles was greater than 1500 or −1500 Newtons per second, respectively [13]. Ten force and time related variables were extracted from the measurements. Two parameters were calculated from the first third (IC to 33%) and eight parameters from the entire (IC to TO; 100%) stance phase (Table 1). Finally, the FSA was identified for each IC captured. To achieve this, raw kinematic data were filtered using a low-pass 15 Hz filter. Visual 3D ×64 Professional (v6.03.06; Germantown, MD, USA) was used to model the foot segment so that the shoe-elicited angulation was negated and the subsequent foot segment angle (in relation to the laboratory coordinate system) was reported [39].

### 2.4. Modeling Approaches

As a pre-requisite for model development, all variables were assessed for normality (i.e., skewness or kurtosis statistic ≤ 2.58). If the assumption of a normal distribution was not met for any of the variables, a natural logarithm transformation was performed to ensure their use was appropriate for parametric statistics (noted in Table 1). The data was then split record-wise into two sets; one was a training data set (70%; n = 2442 steps) and the other a validation (“test” or “hold out”) set (30%; n = 1047 steps). This was done to avoid model under-fitting and high classification errors [40,41,42].

Three modelling techniques (multiple linear regression, conditional inference tree and random forest) were then trained using the training data set to predict FSA and to classify FSP from the pressure insole data. For the classification of FSP, all models employed the degree-based ranges defined by Altman and Davis [14] to categorize steps into either FF (FSA < −1.6°), MF (−1.6° ≤ FSA ≤ 8.0°), or RF (FSA > 8.0°). Steps were classified regardless of the trial condition in which they were performed (i.e., the extreme FF and FF conditions were primarily classified as FF strikes, and similarly, extreme RF and RF conditions as RF strikes).

### 2.5. Model Development

First, a parametric stepwise multiple linear regression (MR) to predict the FSA at IC was modelled using SPSS Statistics (SPSS Inc.; Version 26.0, Chicago, IL, USA). Seven significant (α = 0.05) regression equations were developed (F-to-enter ≤ 0.050, F-to-remove ≥ 0.0100), therefore the Akaike Information Criterion (AIC) and Schwartz-Bayesian Information Criterion (BIC) were calculated for each regression to guide model selection for the subsequent comparisons [43]. The resulting model (Equation (1)) retained the lowest AIC and BIC, and highest model fit (R^2^ = 0.914, R^2^_ADJUSTED_ = 0.914; *p* < 0.001; standard error of the estimate = 5.10°; df = 2434). The same MR model was used for classification by categorizing the predicted FSA (calculated from Equation (1)) of the validation set according to the previously mentioned FSP ranges [14].

(1) $$FSA=-\;89.2+94.4\;\left(IV_{1}\right)+62.3\;\left(IV_{2}\right)+17.9\;\left(IV_{3}\right)+\;8.8\;\left(IV_{4}\right)-8.4\;\left(IV_{5}\right)+3.4\;\left(IV_{6}\right)+1.8\;\left(IV_{7}\right)$$

where $IV_{1}$ = IR_Aft, $IV_{2}$ = PF_Fore, $IV_{3}$ = RFD_Aft, $IV_{4}\;$= IR_Aft_0-33%_, $IV_{5}\;$= PF_Aft, $IV_{6}\;$= Ln(%RFD_Fore), $IV_{7}\;$= Ln(%RFD_Aft).

Two conditional inference trees were modeled with the statistical software R (“ctree” function of “partykit” package) [34,44,45]. The two models differed in their outcomes: one model predicted continuous FSA (TREE_PRED_), while the other classified FSP (TREE_CLASS_; defined classes: RF, MF, FF). For both models, the significance level was set to α = 0.01 (minimum splitting criterion = 0.99). A maximum depth of eight was achieved for TREE_PRED_, and TREE_CLASS_ achieved a depth of six.

**Figure 2:**
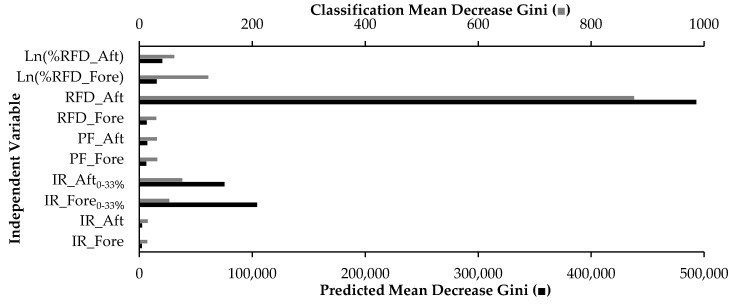
The variable importance for the random forest model of foot strike pattern classification is presented with gray bars (scaled to the secondary *x*-axis), while the foot strike angle prediction is presented in black (primary *x*-axis). The variables of higher importance can be seen with larger “Mean Decrease Gini.”.

Finally, two random forest models as developed by Breiman [35] were trained using the statistical software R (“randomForest” package) [44,46]. The first model was trained for the purpose of continuous FSA prediction (FRST_PRED_) and the second for FSP classification (FRST_CLASS_). A large number of trees (n = 500) was selected for the development of the FRST_PRED_ and FRST_CLASS_ models to decrease out-of-bag errors [47]. Variable selection was randomly initialized in order to define candidates for each split. The final models were chosen because they had the lowest root mean squared error (RMSE; FRST_PRED_) and the highest mean accuracy (FRST_CLASS_) in a 5-fold cross-validation comparison of the different parameter settings (“caret” package) [44,48]. The important variables for the FRST_PRED_ and FRST_CLASS_ can be seen in Figure 2, where high “Mean Decrease Gini” is associated with decreased node impurity, and therefore higher variable importance [49].

### 2.6. Model Accuracy and Precision

The models for FSA (MR, TREE_PRED_, FRST_PRED_) and FSP (MR, TREE_CLASS_, and FRST_CLASS_) were tested with the remaining validation set (n = 1047 steps). Accuracy and precision metrics were calculated for each of the models using the comparison of the true FSA/FSP (measured with 3D kinematics) and the estimated FSA/FSP (i.e., estimated from Loadsol^TM^ metrics).

For model comparison of the three approaches that predicted FSA (MR, TREE_PRED_, and FRST_PRED_), four performance metrics were calculated per recommendations of Galdi and Tagliaferri [50]. These included the mean squared error (MSE), RMSE, mean absolute error (MAE) and mean absolute percentage error (MAPE) of the true versus predicted FSA outcomes. The precision of the prediction models was quantified by calculating the limits of agreement (LoA) and bias of the predicted data set according to Bland and Altman [51]. Specifically, the 95% LoA was calculated using the mean difference (true FSA–predicted FSA) ± 1.96 standard deviations of the differences, and the maximum precision was reported as the difference between the subsequent limits.

Confusion matrices were created for the FSP classification models (MR, TREE_CLASS_, and FRST_CLASS_) utilizing the true classes (measured by kinematic FSA) and the estimated classes (i.e., the class estimated from each model). From these confusion matrices, three metrics were computed as recommended by Galdi and Tagliaferri [50] for model comparison. These included the model accuracy (Equation (2)), classifier recall (Equation (3)), and classifier precision (Equation (4)).

(2) $$model\;accuracy\;=\;\;\frac{total\;correct\;}{total\;sample\;\left(n\right)}\times 100\%$$

(3) $$classifier\;recall\;=\;\;\;\frac{true\;positives\;of\;a\;true\;class\;}{total\;sample\;of\;a\;true\;class}\times 100\%$$

(4) $$classifier\;precision\;=\;\;\;\frac{true\;positives\;of\;a\;estimated\;class}{total\;sample\;of\;a\;estimated\;class}\times 100\%$$

where total correct = number of cases correctly classified, true class = true positives + false negatives of a classifier, estimated class = true positives + false positives of a classifier

## 3. Results

Descriptive statistics (mean ± standard deviation) are presented in Table 2 for each of the independent variables of each step according to their FSP class (FF, MF, RF).

### 3.1. FSA Prediction

**Figure 3:**
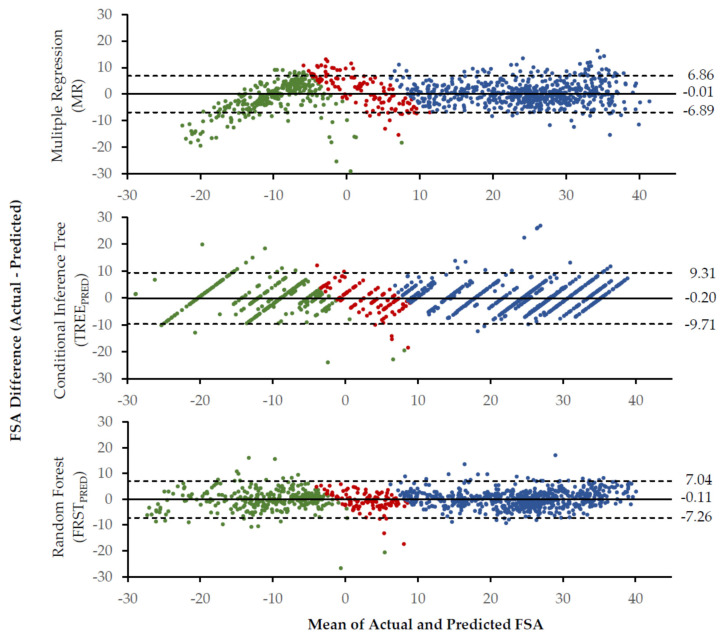
The Bland–Altman bias (solid line) and 95% limits of agreement (dashed lines) are presented for each of the foot strike angle (FSA) prediction methods. Green = rear foot strikes; Red = mid foot strikes; Blue = fore foot strikes; Bias = average of the residuals; Limits of agreement = ± 1.96 standard deviations around the bias.

The Bland–Altman Bias and Precision of the FSA prediction models is shown in Figure 3. Further FSA prediction model accuracy can also be seen in Table 3. In general, the FRST_PRED_ performed with greater prediction accuracy than the MR or TREE_PRED_. The MR and FRST_PRED_ had minimal biases (MR = −0.01, FRST_PRED_ = −0.11; Figure 3) and the maximum precision of the two methods was less than 15° (MR = 13.75°, FRST_PRED_ = 14.30°). A larger maximum precision was found for the TREE_PRED_ (19.02°).

### 3.2. FSP Classification

The confusion matrices developed for each FSP classification model (MR, TREE_CLASS_, FRST_CLASS_) are displayed in Table 4A. The associated accuracy (Equation (2)), recall (Equation (3)), and precision (Equation (4)) results are presented in Table 4B. All models yielded classification accuracies larger than 90% (Table 4B). The MF condition had markedly lower recall and precision than its RF and FF counterparts for all models calculated (Table 4B).

## 4. Discussion

The purposes of the current study were to compare three statistical techniques used to (i) predict FSA and (ii) classify FSP using independent variables derived from the Loadsol^TM^ pressure insoles. Generally, clear differences in the three foot strike styles were noticeable by similarly stratified independent variables (Table 2), with the exception of the variable PF_Fore. For this variable, the differentiation between FF and MF strike types is not clear. This lack of dichotomy may be a result of speed or flight time inconsistencies during MF strike pattern performance, which is supported by the fact that the MF condition was the most difficult condition for participants to perform [37]. However, the apparent stratification of the independent variables for each strike condition thus confirms the applicability of the fore/aft Loadsol^TM^ sensors to estimate FSA and FSP [26,27]. Supporting this, the MR and FRST_PRED_ models developed for the prediction of FSA were both evidently good fits (MR = 91.4% and FRST_PRED_ = 95.42% of variance explained) and the classification accuracy of FSP for all statistical techniques was greater than 90% (Table 4B).

### 4.1. FSA Prediction

The three models (MR, TREE_PRED_ and FRST_PRED_) assessed for FSA prediction had comparable performance when tested using the validation set (Figure 3 and Table 3). The most important independent variable in the FRST model was RFD_Aft as evidenced by the highest mean decrease in node impurity (Gini; Figure 2). Importantly, RFD_Aft is also a predictor used in the TREE_PRED_ and MR models. However, the specific variable importance of RFD_Aft in the MR (via beta coefficients) cannot be interpreted because the model violated the assumption of collinearity [52]. Collinearity considered, the overall prediction of the model should be unaffected [52].

**Figure 4:**
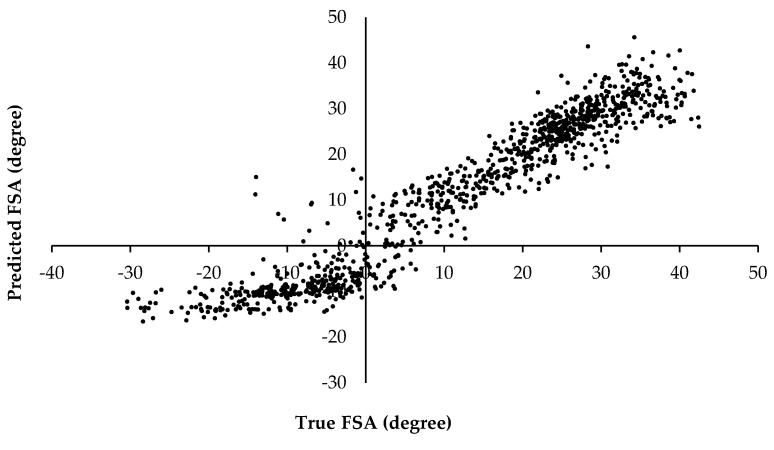
The relationship between the true foot strike angle (FSA) and that predicted by the multiple regression is shown for the distribution of the foot falls included in the study. The strike patterns can be discerned from the following scale: fore foot: FSA < −1.6°; mid foot: −1.6° ≤ FSA ≤ 8.0°; rear foot: FSA > 8.0°.

The linear approach of the MR as suggested by Fritz and colleagues [27] appears to be appropriate to generally explain the variance of the FSA (R^2^ = 0.914). In a similar application, a univariate linear regression to determine strike index via the onset time difference of a fore and aft pressure sensor resulted in a lower coefficient of determination (R^2^ = 0.836) [26]. Although participants were asked to perform RF, MF, and FF foot strikes, Cheung and colleagues [26] did not carry out further analyses to confirm the performance of the FSPs or if there was a stratified model fit. Importantly, a strong linear relationship between the strike index and 3D FSA kinematics is supported in literature, however the relationship appears to be driven primarily by FF and RF strike types [14,16]. Upon visual inspection, those foot strikes that fell closer to the MF range of FSA had the largest standard errors [16]. A similar visual phenomenon is seen in the current study’s data, however the more extreme FF and RF also appear to be indicative of greater prediction errors (Figure 3 and Figure 4). The methodological inclusion of the extreme FF and RF conditions in the current study make it possible to see the potential that there are two linear relationships (Figure 4). Thus, greater accuracy in FSA prediction using MR may be gained from developing a model for the RF and FF FSPs independently.

Importantly, the midfoot and more extreme RF strikes are not as well predicted by the MR than by the FRST_PRED_ (Figure 3). However, both models exhibit higher numbers of residuals outside of the Bland–Altman limits of agreement at the extremes of FF foot strike pattern. Additional proportional bias may be evidenced in the extreme FF range of the MR. However, because these extreme foot strike patterns were considered “exaggerated” to the participants (as was their instruction), the bias present there may not influence the practical application of such models. Further, the stratification seen in the TREE_PRED_ Bland–Altman makes it apparent that it’s use for continuous FSA prediction is limited to the number of outcomes (i.e., maximum tree depth) included in the model (Figure 3). Ultimately, the TREE_PRED_ appears to be better suited for discrete classification problems, whereas the FRST_PRED_ is arguably the most appropriate model for prediction problems that include a large range of FSAs or number of MF strikes.

### 4.2. FSP Classification

Although the overall classification accuracy of the MR was greater than 90%, the MF strike was only properly classified with 38% recall (Table 4). Conversely, TREE_CLASS_ and FRST_CLASS_ classified the MF strike with approximately 73% and 75% recall (Table 4). This is similar to the findings of Delgado-Gonzalo and colleagues [53], who found that the MF condition was classified with the least recall and precision using accelerometer-based inputs. Importantly, the MF strike pattern in the current study may have been classified with the least accuracy because it had the least number of samples in the training set (MF = 197, RF = 1495, FF = 650). Supporting this theory, the RF pattern classified with the highest recall in the MR and TREE_CLASS_ methods (98–99%) and was the greatest sample contributor in the training set. Further, the most important variable for the FRST_CLASS_ was RFD_Aft (Figure 2), which is consistent with the first splitting node variable of TREE_CLASS_. The models may be best suited to distinguish between RF and FF strikes primarily due to the lack of independent variable or sensor differentiation regarding the middle region of the foot. Thus, a three-part sensor insole that highlights the central region of the foot (thus allowing a variable such as the mid-region rate of force development) may be better suited for MF classifications. However, Lieberman and colleagues [9] found that habitually shod runners primarily perform RF strike patterns, therefore the current models should serve recreational runners well.

For populations of shod runners who have consciously altered or retrained their running foot strike pattern (i.e., those investigated by Cheung and Davis [11]), the higher accuracy of the FRST_CLASS_ may provide further confidence in the MF classifications. However, the future use of simple methods like the MR or TREE_CLASS_ methods should not be discounted because equal class sizes in the training set may improve the recall of MF classifications and overall model accuracy.

### 4.3. Application

The results of the current study support that a two sensor (fore and aft) pressure insoles can be used to predict and classify foot strike with sufficient accuracy. Compared to previous works with the aim of estimating FSA using IMU sensors [15], the current results boast lower bias when compared to a reference 3D motion capture camera system (FRST_PRED_ of current study = −0.11° vs. IMU = 3.9°) and only slightly worse precision (FRST_PRED_ of current study = 14.30° vs. IMU = 10.6°). This raises the potential of an insole sensor to provide the holistic pairing of kinetic and kinematic information regarding performance and injury indicators during running. An ankle joint torque MR prediction model has already been developed with adequate accuracy (R^2^_ADJUSTED_ = 0.831, RMSE = 6.91 Nm) using the independent input of 99-sensor pressure insoles [54]. Further, vertical GRF from pressure insoles have been used to predict the 3D GRF components using MR and Artificial Neural Networks, supporting that power and injury related variables can be considered a possibility via simple wearable sensors [55]. From an application standpoint, although many independent variables are used in the models of the current study, they all are derived from a single system. The use of a single system thus reduces the necessity of the synchronization and additional processing power of a supplementary system. A larger range of running conditions could be studied in the future, which may allow for the reduction of independent variables and further encourage the potential to transition toward “real time” foot strike pattern and angle detection.

The current study thus lays the framework for FSA and FSP detection in insoles with larger numbers of sensors (like those used by Billing et al. and Fong et al. [54,55]). This framework may be useful in the push to define and detect running power accurately. The calculation of power during running is a controversial topic due to the complexity of the human biomechanical system, and many of the current commercial systems do not have proven validity in calculating the metric [56]. Therefore, a kinetic approach may exceed current IMU-based calculation methods (i.e., Stryd^TM^ foot pods) due to the immense information a multi-sensor pressure insole can provide.

## 5. Conclusions

The current study supports the feasibility of two-sensor pressure insoles to detect FSA and FSP, and therefore aids in the research and coaching of running movements, as well as consumer-based shoe prescription. Simple machine learning techniques can be used to predict and classify runners’ foot strike patterns with accuracies greater than 90%. However, foot falls that are a true MF strike are incorrectly classified more often than RF or FF strikes by these methods. A greater accuracy can be accomplished with the application of a more complex machine learning technique like a FRST. The current study was limited in its collection of MF steps, therefore more MF steps or using over- or under-sampling techniques may improve the classification of the MF pattern in the future. Further, the machine learning techniques should be applied to running with higher ecological validity that encompasses variable metabolic intensities (i.e., speeds), and limited changes of direction.

## Figures and Tables

**Table 1:** Ten variables calculated from the Loadsol^TM^ insole measurements are defined with respect to the sensor used and the percentage of the stance phase used in calculation.

Parent Variable	Variable	Definition	Insole Sensor	Stance Phase [%]	Abbreviation
Impulse [N∙s]	Impulse Ratio [%]	Impulse ratio between the insole sensor and total foot during the entire or first third of the stance phase	Fore	0–100%	IR_Fore
			Aft	0–100%	IR_Aft
			Fore	0-33%	IR_Fore_0-33%_
			Aft	0-33%	IR_Aft_0-33%_
Peak Force [N]	Peak Force Ratio [%]	Ratio of peak force measured from the insole sensor and total foot during the entire stance phase	Fore	0–100%	PF_Fore
			Aft	0–100%	PF_Aft
Peak RFD [N∙s^−1^]	Peak RFD Ratio [%]	Ratio of peak RFD between the insole sensor and total foot	Fore	0–100%	RFD_Fore
			Aft	0–100%	RFD_Aft
	Ln(Peak RFD) [unit]	Natural logarithm of the occurrence of the peak RFD (as a stance phase %)	Fore	% of Stance	Ln(%RFD_Fore)
			Aft	% of Stance	Ln(%RFD_Aft)

RFD = rate of force development; FF = fore foot; RF = rear foot; N = Newton; s = second.

**Table 2:** Descriptive statistics (mean ± standard deviation) are presented for each variable used in model development, grouped by FSP (classified by measured kinematic FSA).

Variable	Units	FF	MF	RF
FSA	°	−10.2 ± 6.6	3.0 ± 2.8	24.9 ± 8.0
IR_Fore	%	96.2 ± 5.7	89.3 ± 7.0	65.4 ± 11.5
IR_Aft	%	3.8 ± 5.7	10.6 ± 7.0	34.6 ± 11.5
IR_Fore_0-33%_	%	92.5 ± 9.8	77.2 ± 12.9	31.7 ± 16.3
IR_Aft_0-3__3%_	%	7.5 ± 9.9	22.8 ± 12.9	68.2 ± 16.3
PF_Fore	%	95.8 ± 8.2	93.3 ± 6.1	77.0 ± 11.5
PF_Aft	%	8.1 ± 12.3	22.2 ± 13.4	59.9 ± 15.3
RFD_Fore	%	88.3 ± 12.8	70.0 ± 20.8	49.2 ± 16.2
RFD_Aft	%	14.5 ± 16.5	40.5 ± 22.0	91.2 ± 11.0
Ln(%RFD_Fore)	unit	2.69 ± 0.55	2.27 ± 0.33	2.43 ± 0.23
Ln(%RFD_Aft)	unit	2.72 ± 0.41	2.89 ± 0.35	3.25 ± 0.26

**Table 3:** Foot strike angle prediction model performance accuracy is displayed.

	Multiple Regression (MR)	Conditional Inference Tree (TREE_PRED_)	Random Forest (FRST_PRED_)
MSE	26.61	23.57	13.31
RMSE	5.16	4.85	3.65
MAE	3.85	3.51	2.69
MAPE	0.32	0.45	0.33

MSE = mean squared error; RMSE = root mean squared error; MAE = mean absolute error; MAPE = mean absolute percent error.

**Table 4:** Confusion matrices are displayed to indicate where correct (white) and incorrect (grey) classifications occurred for three types of classification methods (multiple linear regression, conditional inference tree, and random forest). Matrices are reported for the validation data set that was not included in model training. All models classified foot strikes into three classes: RF = rear foot, MF = mid foot, and FF = fore foot.

A			Multiple Regression (MR)				Conditional Inference Tree (TREE_CLASS_)					Random Forest (FRST_CLASS_)		
	True	RF	621	13	0	RF	613	21	0		RF	611	23	0
		MF	26	46	48	MF	14	88	18		MF	16	92	12
		FF	5	8	280	FF	5	6	282		FF	3	8	282
			RF	MF	FF		RF	MF	FF			RF	MF	FF
			Estimated				Estimated					Estimated		
						
**B**						**MR**			**TREE_CLASS_**		**FRST_CLASS_**			
Accuracy (%)				ALL		90.4			93.9		94.1			
Recall (%)				RF		97.9			96.7		96.4			
				MF		38.0			73.3		76.7			
				FF		95.6			96.3		96.3			
				RF		95.2			97.0		97.0			
Precision (%)				MF		68.7			76.5		74.8			
				FF		85.4			94.0		95.9			
